# Complementary feeding in Kongwa, Tanzania: Findings to inform a mycotoxin mitigation trial

**DOI:** 10.1111/mcn.13188

**Published:** 2021-05-04

**Authors:** Clara Mollay, Neema Kassim, Rebecca Stoltzfus, Martin Kimanya

**Affiliations:** ^1^ Department of Food Biotechnology and Nutritional Sciences, School of Life Sciences and Bioengineering Nelson Mandela African Institution of Science and Technology Arusha Tanzania; ^2^ Goshen College Goshen Indiana USA

**Keywords:** aflatoxin, complementary feeding practices, fumonisin, infant and young child, Tanzania, undernutrition

## Abstract

Complementary feeding of 6‐ to 24‐month‐old infants and young children with adequate, safe and developmentally appropriate food is essential to child health. Inappropriate complementary foods and feeding practices are linked to the high incidences of undernutrition among infant and young children in most developing countries, including Tanzania. Mycotoxin risk is an additional concern, given the documented presence of aflatoxin and fumonisin in food systems of Africa, especially maize and groundnut. In preparation for a trial of mycotoxin mitigation, we conducted focus group discussions and recipe trials to explore complementary foods and feeding practices in Kongwa, a rural district of central Tanzania. Sixty mothers of infants from 6 to 18 months of age in five villages across the district were purposefully sampled. During focus group discussions, mothers reported to mostly feed their children with cereal and groundnut‐based foods as thin or thick porridges. The most common porridge preparations contained cereal (mostly, maize) ranging from 66.7% to 80.0% by weight and groundnuts from 7.7% to 33.3%. The ratio of cereal to groundnut ranged from 3:1 to 4:1. For the recipe trial sessions, mothers chose similar ingredients reported during discussions to prepare complementary foods. The reliance on maize and groundnuts in complementary foods predisposes the children to undernutrition and exposure to aflatoxins and fumonisins. These formative research results suggest multiple intervention points to improve complementary feeding and reduce mycotoxin exposure in this population, including education messages package on feeding practices, mycotoxin control practices and complementary food formulation.

**Key messages**

- Maize and groundnuts (which are commonly contaminated with aflatoxins and fumonisins) are the main components of complementary foods in Kongwa District.
- The reliance on maize and groundnuts as main components of complementary foods increases the risk of undernutrition and exposure to aflatoxins and fumonisins among IYC of Kongwa.
- Educating the mothers or caregivers on appropriate complementary foods and feeding practices is needed to improve nutritional status and minimize the risk of dietary exposure to aflatoxins and fumonisins in this community.

## 1 INTRODUCTION

Exclusive breastfeeding in the first 6 months of life (Butte et al., 2002; Kramer & Kakuma, 2001) and appropriate complementary feeding from around 6 months of age are critical for early childhood growth and development (WHO/PAHO, 2003). After exclusive breastfeeding for the recommended period of 6 months, complementary feeding of 6‐ to 24‐month‐old infants and young children (IYC) with safe, nutritious and developmentally appropriate food is imperative to prevent undernutrition and exposure to food‐borne toxins and to promote healthy growth and development (Watson et al., 2017; WHO, 2008; WHO/PAHO, 2003).

Widespread undernutrition in low‐ and middle‐income countries continues to exert enormous costs in terms of the survival of IYC (Bhutta et al., 2008; Victora et al., 2008). Undernutrition is linked to stunted growth, delayed cognitive development, late entry into school, decreased schooling years, reduced school achievement, reduced economic productivity in adulthood and poor maternal reproductive outcomes (Crookston et al., 2010; Dewey & Begum, 2011; Mayneris‐Perxachs & Swann, 2019).

Stunting and micronutrient deficiencies are common in Tanzania. According to the 2015–2016 Demographic and Health Survey in Tanzania (MoHCDGEC et al., 2016), the prevalence of stunting among children aged 6–59 months was 34% in 2015–2016. The 2015–2016 survey further showed that only 8% of 6‐ to 23‐month‐old IYC receives the minimum acceptable diet as defined by the World Health Organization (WHO) (MoHCDGEC et al., 2016; WHO, 2008).

Inappropriate complementary feeding practices are linked to high incidences of undernutrition among the IYC in Tanzania (Victor et al., 2014) and are closely associated with the education status of mothers and limited access to health services (Ogbo et al., 2018).

The presence of mycotoxins in complementary foods may further contribute to poor child growth (Anitha et al., 2020; Kimanya et al., 2010). According to Tola and Kebede (2016), mycotoxins are harmful secondary metabolites of certain fungi that contaminate foods like cereal crops, leguminous plants and animal feeds and products, with negative impacts on human and animal health. The two major classes of mycotoxins that contaminate maize are aflatoxins and fumonisins (Bankole et al., 2006; Kimanya et al., 2008, 2014). Various studies have suggested that the exposure to either of these mycotoxins through complementary foods is inversely proportional to child growth (Chen et al., 2018; Gong et al., 2002, 2003, 2004; Shirima et al., 2015; Turner, 2013; Turner et al., 2007). One study found an association between stunted growth in Tanzanian infants and exposure to fumonisins (Kimanya et al., 2010). A high risk of aflatoxins and its co‐contamination with fumonisins in complementary foods in Tanzania has also been reported by Kimanya et al. (2014) and Kamala et al. (2017).

Despite the prevalent stunting, widespread suboptimal feeding practices and vulnerability of foods to mycotoxins in Tanzania, there are limited studies on feeding practices and the perception of mothers around complementary foods in Tanzania. Although governmental and non‐governmental programmes/interventions have been mounted to alleviate child undernutrition, the suboptimal complementary feeding practices persist in Tanzania (Victor et al., 2014; Watson et al., 2017). This study empirically explored mothers' feeding practices, specifically identifying porridge ingredient and recipe preferences; steps of preparation, cooking and feeding consistency and nutrient density; and attitudes and perceptions of feeding groundnuts to IYC to inform the development of an intervention for a future mycotoxin mitigation trial in the Kongwa District of central Tanzania.

## 2 MATERIALS AND METHODS

### 2.1 Study site

This study was conducted in the Kongwa District of Dodoma Region in central Tanzania. Dodoma Region was purposively chosen as a study site not only because of the high stunting rate of 56% and 37% in 2010 and 2016, respectively, but also due to a higher population size compared to other districts in the region (MoHCDGEC et al., 2016; NBS & OCGS, 2013). Moreover, the district is geographically close to Kibaigwa international maize and groundnuts market, thereby increasing the likelihood of access to these foods for use in complementary feeding.

### 2.2 Sampling

Five villages (Ibwaga, Nghumbi, Songambele A, Pandambili B and Mkoka) out of 87 of Kongwa District were selected for the study. Based on discussions with the District Nutrition Officer, the villages were selected to represent rural and urban settings. A purposive sample of 60 respondents (about 12 from each of the five villages) were selected for the study. The respondents were mothers of children aged from 6 to 18 months, identified with guidance from the community health workers (CHWs) in the villages.

### 2.3 Study methods

Exploratory methods described by Dickin and Griffiths (1997) were employed in the study. Focus group discussions (FGDs) and recipe trials (RTs) (involving one group in each of the five villages) were used to obtain extensive views and observations for use as a basis for improved and effective complementary feeding practices. We explored what and how the mothers feed their children in terms of ingredients and forms as well as the reasons for the choices/practices.

In each group or village, data were collected by three researchers using a predesigned discussion guide with open‐ended questions administered to mothers. One person moderated the discussions, whereas two others took notes. Each village was studied in a single day, with the FGDs conducted first, followed by the RTs. Before the study day, the mothers in each village were asked to bring all items, ingredients and kitchen utensils, which they typically use to prepare complementary feeding porridges or feeding their children at home to the village centre (usually the Village Executive Officer's office).

In the FGDs, mothers were facilitated to discuss foods and feeding practices for their IYC. Specifically, they discussed the age at which their IYC were introduced to complementary foods and type of foods used as complementary foods. They also discussed porridge ingredients, preparation, cooking consistency and feeding. During the RTs, mothers were divided into small groups of three to six persons and asked to prepare complementary feeding porridges and feed their children in the way that they normally do at home. Mothers who could not bring ingredients from home were provided with the ingredients they asked for. One group of each RTs was assigned to make porridge from a 3:1 ratio of maize–groundnut flour mix that had been pre‐blended to assess the acceptability of this formulation for the future trial. This formulation was based on the advice of the District Nutrition Officer. Throughout the RTs, researchers asked questions, observed the practices and noted the mothers' comments, explanations and reactions to each other's statements or actions during the cooking process. They also observed and noted the response of children to complementary feeding, the style of feeding or encouragements used, as well as the amounts served and consumed. The researchers also asked questions related to ingredients added to the complementary feeding porridge. The questions included why a particular ingredient was used, the age of the IYC for whom the ingredient was added and whether or not the choices/uses of ingredients varied with seasons. The researchers also observed the consistency of the prepared foods and enquired whether or not the consistencies varied with the age of the IYC. They also observed mothers' feelings about the appearance, smell, consistency and appropriate feeding portion sizes and frequency for babies at a given age. Open‐ended questions were used, and perceptions of groundnut use in complementary foods were raised and discussed. The researchers also observed the consistency and nutrient density of the prepared complementary foods during RT assessment.

### 2.4 Data analysis

The discussions and notes obtained during FGDs and RTs were in Swahili. The transcripts of the responses were entered into Excel sheets and translated from Swahili to English. Codes were created by assigning short phrases to salient data sets that represented important (recurring) themes together with memo writing as described by Corbin and Strauss (2008). Further management and final analysis of data were done in ATLAS.ti Version 7 software (ATLAS.ti. Scientific Software Development GmbH) (Friese, 2016) in which the results were organized using the determined final themes and quotations.

### 2.5 Ethical considerations

This study was approved by the National Medical Research Institute of Tanzania (NIMR), document number NIMR/HQ/R8a/Vol.IX/2874. Mothers of eligible children signed informed written consent to participate in the study. The permission to conduct the study was also obtained from Kongwa District authorities.

## 3 RESULTS

### 3.1 Common complementary foods

During FGD, most of the mothers reported that they started feeding their infants with complementary foods at 6 months of age. On the other hand, few mothers practised both earlier (at 5, 4 and 3 months) and late (at 8 months) initiation of complementary foods to infants. Tables 1 and 2 show the common complementary foods in each of the study villages. A wide range of complementary foods in the study area was largely maize based and provided in form of typical family food or as a thick or thin porridge. Legumes such as beans or bean soup were given as a part of a meal or occasionally added to complementary porridge. Other complementary foods were groundnuts, Irish potatoes, cow's milk, eggs, fish, sardines, fruits and vegetables. Eggs were mentioned only in Ibwaga village, whereas roots, meat, poultry, yogurt and cheese were not mentioned at all.

**TABLE 1:** The common complementary foods in Kongwa

Village	Main food	Other protein‐based foods	Other foods (fruits and vegetables)	Snacks/others
Ibwaga	Thin porridge, stiff porridge, Irish potatoes	Groundnuts, milk, eggs	Fruits, oranges	Doughnut, biscuits
Nghumbi	Mashed potatoes, thin porridge, stiff porridge.	Milk, sardine sauce	Ripe banana, mango	Artificial juice tea
Mkoka	Thin porridge, banana, potatoes (puree) mashed rice.	Cow's milk, boiled fish, beans soup, sardines	Mangoes, amaranth soup, dried green vegetables, mangoes, watermelon and banana	
Songambele A	Thin porridge, mashed Irish potatoes, stiff porridge banana (puree) with potatoes	Boiled cow's milk	Natural orange juice, mangoes, natural mangoes juice	Artificial mango juice, water, soda
Pandambili B	Thin porridge, stiff porridge, potatoes (puree), thin rice, spaghetti	Cow's milk	Fruits like banana and mangoes	Tea, juice, doughnut
Overall	Thin porridge, stiff porridge, Irish potatoes, thin rice, porridge, banana, spaghetti	Groundnuts, milk, eggs, sardine sauce, boiled fish, beans soup	Oranges, ripe banana, mango, amaranth soup, dried green vegetables, watermelon	Doughnut, biscuits, artificial tea, water, soda

**TABLE 2:** Lishe ingredients

Village	Porridge ingredients	Ingredients weight in kg	Cereals composition in composite flour %^a^	Cereal/groundnut ratio	Groundnuts composition in %^b^	Others (e.g. legumes, sardines or baobab) composition in %^c^
Ibwaga	Finger millet	1	80.0	12:1	7.7	12.3
	Groundnuts	0.25				
	Rice	0.5				
	Soybeans	0.5				
	Maize (whole)	0.528				
	Sorghum	1				
	Finger millet	0.5	80.0	4:1	20.0	‐
	Maize	1				
	Groundnuts	0.5				
	Rice	0.5				
Nghumbi	Maize (whole)	1	80.0	4:1	20.0	‐
	Groundnuts	0.25				
	Rice	0.25	75.0	3:1	25.0	‐
	Groundnuts	0.25				
	Maize	0.25				
	Finger millet	0.25				
Songambele A	Groundnuts	0.216	80.0	4:1	20.0	‐
	Rice	0.314				
	Whole maize	0.264				
	Millet	0.276				
	Maize	0.528	80.0	4:1	20.0	‐
	Finger millet	0.56				
	Groundnuts	0.25				
Mkoka	Maize	1	44.4	2:1	33.3	42.9
	Groundnuts	0.5				
	Sardines	0.75				
	Rice	0.25	50.0	3:1	25.0	40.0
	Beans	0.5				
	Maize	0.5				
	Groundnuts	0.25				
	Wheat	1	50	4:1	20	42.9
	Soybeans	1				
	Groundnuts	0.5				
	Sesame	0.5				
	Rice	0.5				
	Baobab^d^	0.5				
Pandambili B	Maize	1	75.0	3:1	25.0	‐
	Rice	0.5				
	Groundnuts	0.5				
	Maize	1	66.7	2:1	33.3	‐
	Groundnuts	0.75				
	Rice	0.5				
	Finger millet	0.56	75.0	3:1	25.0	‐
	Rice	1				
	Groundnuts	0.5				

^a^ Computed from cereals and all other ingredients including groundnuts.

^b^ From cereals and groundnuts only.

^c^ From cereals and others (e.g. legumes, sardines or baobab) excluding groundnuts.

^d^ Baobab powder was mixed with water to make a juice in which other porridge ingredients were then mixed during porridge preparation.

### 3.2 Ingredients and preparation of *lishe*

The ingredients/materials used in preparation of complementary porridge flour included maize, sorghum, finger millet, rice, sesame, wheat and groundnut. The ingredients/materials were prepared and mixed by the mothers at home and milled at nearby hammer mills as was explored during FGDs. The blended flour is commonly referred to as *lishe*. The mothers reported more than 10 different formulations of *lishe* as shown in Table 2. The most commonly used ingredients were maize and groundnuts. Other less commonly used ingredients are rice, millet, sorghum, finger millet, soya bean, sardines, beans, wheat, baobab and tamarind.

Around 75% of the *lishe* blends contain cereal at proportions ranging from 66.7% to 80.0%, weight by weight. All (100%) of *lishe* contained groundnuts. The proportion of groundnuts in the composition of *lishe* ranged from 7.7% to 33.3%, weight by weight. This information was given by mothers during FGDs. In very rare incidents, other ingredients such as sugar, Blue Band margarine and juice prepared from baobab powder were mixed with the porridge during preparation.

### 3.3 Sources of ingredients

During both FGDs and RTs, the mothers reported purchasing groundnuts and maize during July to December and January to May respectively, when own produced materials are out of stock. Other ingredients such as sugar, *Blue Band* margarine, salt, rice, wheat and soya bean were purchased and used depending on the availability of funds. One of the respondents said: ‘We in most cases use these as ingredients but not all. What we use depends on what we can afford at the moment’.

### 3.4 Lishe preparation knowledge and practices

A majority of mothers knew the importance of cleaning *lishe* ingredients, roasting groundnuts and removing the outer coat as well as drying *lishe* soon after milling to reduce the moisture content. Individual ingredients were cleaned by sorting, removing sands and winnowing. Additionally, soya bean were boiled, washed and then sun‐dried. Occasionally, groundnuts were roasted before were mixed with the other ingredients. However, during the FDGs, we observed that some of the mothers did not sort, winnow or wash cereals (mostly the maize) and groundnuts. A majority of the mothers reported that they sort rice, but we observed that some mothers did not de‐hull cereals like maize or wheat. Some mothers reported storing the milled *lishe* for up to 2 weeks, which may be too long to preserve its safety and quality.

### 3.5 Food consumption and feeding frequency

During FGDs, most mothers reported preparing complementary feeding porridge and feeding their children once to thrice a day on daily basis (7 days/week). The observation made during RTs showed that in each feeding, a child consumed about 1 tbsp to one cup (about 250 mL) of a thin porridge, depending on age.

### 3.6 Preferences, beliefs and perceptions about complementary foods

Most mothers used groundnuts in complementary food flours because they believed that it is nutritious and can enhance child health. The researchers probed the mothers to talk about the thickness of the porridges they made. Some mothers preferred thick to thin porridge because it satisfies some babies, especially the hungry ones. When one mother was asked why she desired a thick porridge for her child, she gave this response: ‘Thick porridge stays in a child's stomach for longer and gives him energy’. However, they also observed that thick porridge does not cook well and, in some cases, babies would vomit when fed on it, whereas the younger infants (6–12 months) mostly refused it. Others preferred thin porridge because it is easier to feed and easier to swallow. When a mother was asked about her preference for thin porridge, she replied, ‘Thin porridge is good, the child prefers it and it is easy to swallow’.

### 3.7 Feeding styles

During the RTs, the mothers were asked to feed their IYC in the actual way they do at home for the researchers to explore the mothers' responsiveness and affection. From the researchers' observations, mothers' feeding practices and styles encouraged the children to eat. Mothers introduced complementary porridge in a cup and fed their infants with spoons. During feeding, a majority of mothers carried their infants on their laps and were observed to encourage them to eat the complementary feeding porridge, in different ways, for instance, by petting them, shaking their hands or saying, ‘Take, this is sweet!’ while looking at them. Besides, some mothers fed their children while they were free to play around whereby they generally accepted and enjoyed the porridge offered to them.

### 3.8 Bad experiences of feeding on groundnuts

Some mothers expressed their belief that groundnuts may contain fungi that can cause diarrhoea and requested to be provided with adequate guidelines and instructions on proper preparation, cooking and storage of *lishe* flour to avoid fungal infection.

Apparently, the mothers had information about the negative effects of feeding children on groundnut‐based porridge, although this was not directly proven during our observation. When a researcher asked mothers if they had heard of any information concerning the health effects of groundnuts, one mother said, ‘When stored for a long time, groundnuts develop fungus and can affect the child’.

Another mother said, ‘Yes, if you add groundnut into your child's food, they will suffer from allergies like skin rashes’.

Nevertheless, regardless of the observed beliefs about groundnuts, the mothers still preferred feeding their children on groundnuts. A researcher asked mothers if they would be ready to feed their children on groundnut‐based porridge if someone suggests so, and all mothers confirmed that they would.

## 4 DISCUSSION

The present study provides an exploratory description of mother's perceptions and practices about porridge ingredients, cooking and feeding techniques and perceptions about groundnuts, maize and other common ingredients for complementary foods in Kongwa.

The research results presented in this paper also reveal that the formulations of complementary porridge flour were home‐made with a wide range of cereals, with maize and groundnuts being the most common ingredients. It was also observed that some formulations had three kinds of cereal (maize, rice and finger millet), one legume (groundnuts), two kinds of cereal (maize and rice) or two legumes (beans and groundnuts).

The RTs revealed that the mothers' practised the authoritative responsive child feeding style (Birch & Fisher, 1995; Hodges et al., 2008). Similar findings were obtained in a cross‐sectional study in Southern Ethiopia (Wondafrash et al., 2012). Our results, however, differ from others such as Kinabo et al. (2017), which showed that caregivers in Unguja Islands, Tanzania, rarely practised responsive feeding, and Dharmasoma et al. (2020), where none of the caregivers in Sri Lanka practised responsive feeding fully. Other studies suggest that the responsive feeding style may contribute to the healthy growth, development and well‐being of children (Black & Aboud, 2011; Daniels, 2019). In contrast, non‐responsive feeding (authoritarian) is associated with an increased risk of overweight and/or obesity in children (Hurley et al., 2011; Vollmer & Mobley, 2013) due to lack of self‐regulated feeding.

The WHO recommends adequate consistency of complementary foods through the gradual increase of food consistency from 6 months onwards (WHO, 2005). The mothers expressed that the right consistency of complementary porridge depended on the age of the child. However, other mothers reported that a thick porridge can choke the child.

The mothers appeared to adhere to similar methods of complementary porridge preparation and feeding their children. The uniformity of methods was notably in terms of ingredients used to prepare complementary porridge with little diversity of food groups.

Our observations suggest that the mothers might have learned some good feeding practices such as the consistency of food given to IYC depending on age from the Mwanzo Bora Nutrition Project (MBNP). The District Nutrition Officer explained that the Mwanzo Bora Project was implemented in the study area before our formative research. Among other objectives, the projects aimed at reducing stunting among children in the Dodoma Region where the present study was located. The interventions provided by MBNP included training the mothers on education packages using its Social and Behaviour Change Communication (SBCC) Kit, the *Mkoba wa Siku 1000*. The kit was developed in collaboration with MBNP and Tanzania Food and Nutrition Centre (TFNC) to influence positive nutrition behaviours and improve maternally and child nutrition.

The findings in this study further indicated that a majority of the mothers initiate complementary foods to their infants at 6 months of age. These findings are contrary to results of the Tanzania demographic and health surveys of 2015–2016 (MoHCDGEC et al., 2016) and various studies in Tanzania that have observed poor timely initiation of complementary foods (de Bruyn et al., 2018; Kinabo et al., 2017; Kulwa et al., 2006; Maonga et al., 2016; Mgongo et al., 2013; Nkala & Msuya, 2011; Shirima et al., 2001). We probed for true behaviours because mothers were well educated on the recommended time for the introduction of complementary feeding that is at 6 months of age. However, few mothers practised both earlier (before 6 months) and late (over 8 months) initiation of complementary foods to infants. These practices are known to be significant contributors to malnutrition in infants as they are associated with inadequate nutrient intake and high infection rates (Sellen, 1998).

The IYC in Nghumbi and Pandambili B villages received less diversified food compared with the other three villages. The low diet diversification was related to local nutrition knowledge in Tanzania. These findings are in line with the observation by Christian et al. (2016) in Ghana and Kuchenbecker et al. (2017) in Malawi that nutrition knowledge and attitude of caregivers may influence children's dietary quality. According to Ochieng et al. (2017), training on food preparation and nutrition can significantly influence dietary diversity in Tanzania. This is important because diversified diets are negatively associated with child stunting and underweight and can ultimately reduce undernutrition in Tanzania (Khamis et al., 2019).

The mothers in the present study did not appear to be aware about mycotoxin contamination in ingredients of *lishe*. Some mothers did not sort maize, groundnuts or other cereals nor de‐hull maize, practices that may influence mycotoxin contamination in complementary feeding porridge (Anitha et al., 2020; Kamala, Kimanya, et al., 2018; Mutegi et al., 2018). However, some mothers seemed to be knowledgeable about the effect of moisture on stored *lishe* and reported to dry the flour before storage. This is in line with results from a qualitative study by Ngoma et al. (2020) in Tanzania where participants were found to be knowledgeable of the effect of mould or aflatoxin contamination of improperly dried complementary food. The knowledge of aflatoxin in this context might be due to the effort made by the Tanzanian government and its international partners in mitigating aflatoxins in food. For instance, from 2017 to 2019, the Food and Agriculture Organization of the United Nations in partnership with the government of Tanzania implemented in Dodoma and Manyara Regions a project on aflatoxin mitigation response through dissemination of appropriate post‐harvest management technologies and awareness raising.

It is well known that complementary foods in Tanzania are cereal based (Kimanya et al., 2010; Kinabo et al., 2017; Kulwa et al., 2015; Muhimbula et al., 2011; Vitta et al., 2016). Such diets have limited nutrient content (Moursi et al., 2008; Vitta et al., 2016) and are also susceptible to mycotoxin contamination. Groundnuts are prone to aflatoxin contamination and maize to both aflatoxin and fumonisin (Agbetiameh et al., 2017; Kimanya et al., 2008).

The central part of Tanzania where the present study was conducted is the site of a recent outbreak of acute aflatoxicosis that claimed the lives of 20 people, including young children. The outbreak was associated with the consumption of groundnuts and cereal‐based foods contaminated with high levels of aflatoxins (10–51,100 ug/kg) and fumonisins (945–12,630 ug/kg) (Kamala et al., 2018).

Therefore, the IYC in Kongwa Tanzania may be at risk of exposure to aflatoxin and fumonisin and stunted growth (Chen et al., 2018; Kamala et al., 2017; Kimanya et al., 2014). Relying on cereal‐based complementary foods might also limit the bioavailability of micronutrients and the attainment of nutrient adequacy, which may altogether impact negatively on the nutritional status of children during their critical period of growth (Makori et al., 2017).

## 5 CONCLUSION

The findings from this formative research enabled us to identify specific complementary feeding practices that likely contribute to nutrient inadequacy and lead to chronic undernutrition problems in rural central Tanzania. Although the mothers expressed knowledge of the appropriate age to introduce complementary foods as 6 months and the appropriate porridge consistency, the porridges they prepared were made from cereals with largely maize and groundnuts content, which may also lead to the risk of undernutrition and co‐exposure to aflatoxins and fumonisins. In this context, provision of multiple intervention points that include but not limited to education messages packages on feeding practices, mycotoxin control practices and complementary food formulation to improve complementary feeding and reduce mycotoxin exposure in this population is crucial.

## CONFLICTS OF INTEREST

The authors declared that they have no conflicts of interest.

## CONTRIBUTIONS

CM, NK, RS and MK designed the study. CM participated in supervision of fieldwork, data collection and data analysis and wrote the initial and subsequent drafts of the manuscript. MK and RS critically reviewed the manuscript. All authors have agreed to publication of the manuscript.

## Data Availability

The data that support the findings of this study are available from the corresponding author upon reasonable request.
